# Ambient Pressure Drying for Fabrication of Cellulose-Based Lightweight Porous Materials: A Review

**DOI:** 10.3390/gels12070632

**Published:** 2026-07-15

**Authors:** Zhiqiang Xu, Li Wang

**Affiliations:** School of Chemistry and Molecular Engineering, Nanjing Tech University, Nanjing 211816, China; 202321039085@njtech.edu.cn

**Keywords:** cellulose, lightweight porous materials, aerogels, foams, ambient pressure drying, pore collapse

## Abstract

The depletion of fossil resources and environmental pollution issues have accelerated the development of biomass-based lightweight porous materials. Cellulose-based lightweight porous materials (CLPMs) have emerged as ideal alternatives to petroleum-based porous materials owing to their advantages of renewability and biodegradability. Ambient pressure drying has become an ideal solution for the large-scale production of CLPMs; owing to its low cost, short processing time, and ease of operation. However, challenges such as strong capillary forces, reorganization of intermolecular hydrogen bonds, and insufficient framework strength can lead to the collapse of the porous structure during the drying process. This review systematically summarizes the research progress of ambient pressure drying techniques for CLPMs, providing an in-depth analysis of the intrinsic mechanisms underlying structural collapse in terms of capillary pressure, hydrogen bond reorganization, and structural strength. Based on this analysis, five major control strategies for ambient pressure drying, such as solvent substitution and surface hydrophobic modification, are outlined. Furthermore, this review comprehensively discusses the practical application prospects of such materials in adsorption, thermal insulation, and other fields. This review aims to provide theoretical references for the development and industrialization of CLPMs.

## 1. Introduction

Currently, the depletion of fossil resources and environmental pollution have become increasingly acute. At the same time, driven by the global concept of sustainable development, the development of new, renewable, biodegradable, and environmentally friendly materials has become a hot topic in the field of materials science [1]. Due to their unique three-dimensional network structure, porous materials possess low density, high specific surface area, and excellent adsorption and mass-transfer properties, playing an irreplaceable role in numerous fields such as thermal insulation [2,3], catalytic reactions [4,5], environmental remediation [6,7], energy storage [8], and medicine [9,10]. Traditional lightweight porous materials are primarily made from petroleum-based polymers and inorganic materials [11,12], which suffer from drawbacks such as non-biodegradability, high energy consumption during production, and a heavy environmental burden. For example, petroleum-based materials like polystyrene (PS) and polyurethane (PU) foams generate plastic pollution from their waste, which has become a global environmental challenge. Biomass-based porous materials, made from renewable biomass resources (such as cellulose [13], alginates [14,15], and chitin [16,17]), combine the structural advantages of traditional porous materials with biodegradability and are regarded as an ideal alternative to petroleum-based porous materials [18]. Among these, CLPMs have become a research focus in the field of biomass-based porous materials due to their outstanding advantages, including a wide range of raw material sources (wood [19], crop straw [20], sugarcane bagasse [21], cotton [22]), stable chemical structure, tunable mechanical properties and high functionalization potential.

At present, the main types of lightweight and porous cellulose-based materials are cellulose-based aerogels and cellulose-based foams. Both typically exhibit structural characteristics such as high porosity, high specific surface area, and ultra-low density. Their low density and high porosity confer excellent thermal insulation properties, while also providing good adsorption capacity and mechanical stability, demonstrating significant potential to replace traditional materials in various fields [23,24]. Compared to inorganic porous materials, CLPMs exhibit superior mechanical properties; compared to petroleum-based porous materials, their biodegradability and renewability better align with sustainable development needs, making them an environmentally friendly, multifunctional material. The preparation process of CLPMs involves two core steps: (i) preparation of a gel or wet foam from a dispersion, and (ii) drying of the gel or wet foam [25]. The drying process is a critical step that determines the material’s final structure and properties; its primary objective is to remove the solvent from the gel while maximizing the retention of the three-dimensional porous network structure. Currently, the mainstream drying technologies include three categories: freeze-drying, supercritical drying, and ambient pressure drying. Freeze-drying and supercritical drying are associated with high energy consumption, expensive equipment, long production cycles, and high costs, making large-scale production difficult and limiting their industrial application [26,27,28].

Ambient pressure drying, as a low-energy, low-cost, and easily scalable drying technology, holds significant potential for industrial applications; however, its greatest challenge lies in the collapse of the gel network during the drying process. When the solvent evaporates under ambient pressure, the pore structure shrinks and collapses, reducing the material’s porosity and specific surface area [25]. Furthermore, the hydrogen bonding between cellulose molecules undergoes restructuring during the drying process, further aggravating the collapse [28]. Overcoming network collapse during ambient pressure drying to achieve low-energy manufacturing of high-performance CLPMs has become a central research topic in this field. Existing reviews can be broadly categorized into three types, and there are notable gaps in the research. The first type focuses solely on the overall development of cellulose aerogels or foams, broadly covering freeze-drying and supercritical drying, but rarely providing an in-depth analysis of the collapse mechanisms associated with ambient pressure drying. The second type centers on lightweight porous biomass materials, emphasizing processing, modification, and application expansion, but fails to systematically compare how different drying strategies affect pore structure and does not treat cellulose materials as the core research subject. The third type only broadly discusses general strategies for ambient pressure drying of various materials; it does not analyze the causes of collapse in light of cellulose’s unique network system, nor does it summarize targeted solutions for CLPMs. Overall, few reviews have addressed the causes of structural collapse during ambient pressure drying of CLPMs or summarized related drying strategies, leaving important research gaps.

Given the limitations of existing literature reviews, this review adopts a differentiated research perspective, focusing exclusively on ambient pressure drying systems for CLPMs; it provides a critical and comprehensive review of the mechanisms for inhibiting pore collapse, primarily covering cellulose aerogels and cellulose foams, while excluding non-cellulose-based porous materials. Systems containing small amounts of lignin or mixed biomass are briefly mentioned only when they serve as supplements or controls for cellulose-based systems. This review focuses on elucidating the complete mechanisms of collapse in the three-dimensional cellulose network during ambient pressure drying, ranging from microscopic hydrogen bonding to macroscopic pore structure. At the same time, it summarizes the mechanisms of existing mainstream anti-collapse strategies for mitigating capillary tension-induced framework contraction and critically analyzes the advantages and disadvantages of each strategy; moreover, this study summarizes the current status of CLPM applications across various fields, the key bottlenecks facing industrial implementation, and viable future pathways for breakthroughs. By integrating relevant research findings, this review aims to clarify the current strengths and weaknesses of this technology, provide a theoretical basis and technical insights for future research, and promote the low-energy, green, and large-scale development of CLPMs.

## 2. Structural Characteristics of Cellulose

**Figure 1:**
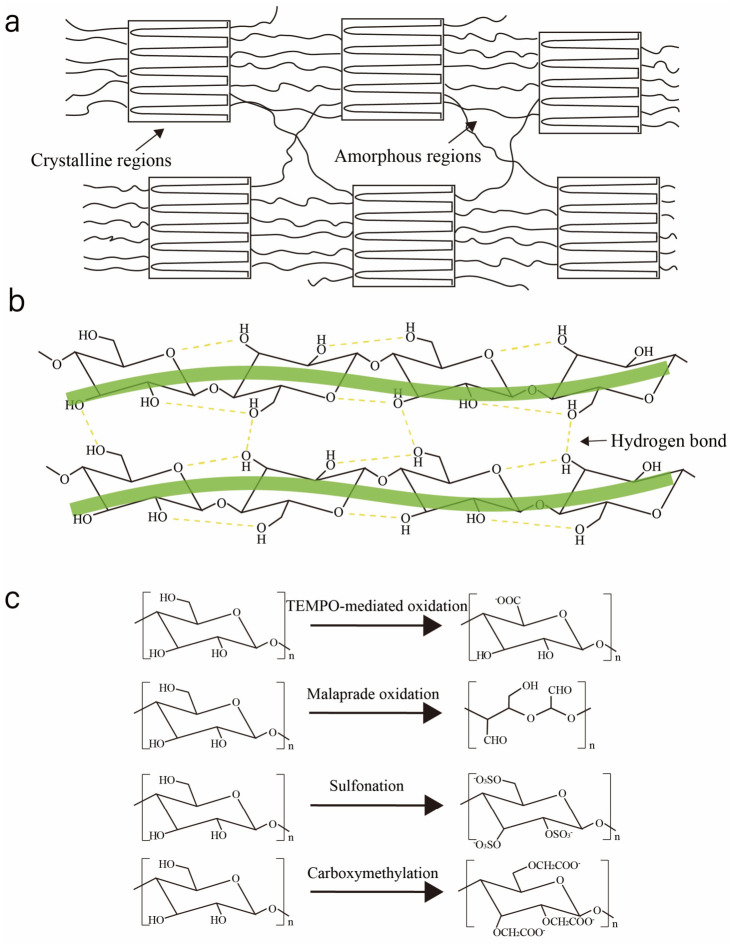
(**a**) Schematic diagram of crystalline and amorphous regions in cellulose. (**b**) Hydrogen bond network in cellulose. (**c**) Common reactions of cellulose.

Cellulose is a linear polysaccharide formed by β-D-glucose units linked via β-1,4-glycosidic bonds [29]. As one of the most abundant and widely used biomass polymers in nature, the numerous hydroxyl groups on its molecular chains can form a multi-level hydrogen bonding network [30]. Crystallinity, hydroxyl content, fiber morphology, and chemical modifiability are the core structural factors that determine the extent of framework collapse, the stability of the three-dimensional network, and the final porous structure of cellulose-based materials during ambient pressure drying. In terms of structural characteristics, cellulose is typically semicrystalline, consisting of both crystalline and amorphous regions (Figure 1a). Crystallinity directly influences the degree of ordered arrangement of molecular chains, the rigidity of the framework, and the sensitivity of the network to shrinkage during drying: the highly ordered chain arrangement and dense hydrogen bond network within crystalline regions help maintain structural stability, whereas amorphous regions are more prone to rearrangement and collapse under the effects of solvent removal and capillary pressure [31,32]. Specifically, in crystalline regions, molecular chains are arranged in parallel and orderly patterns, with uniform and dense hydrogen bonds and strong interchain bonding; highly crystalline cellulose has a stiffer framework that can resist compression from capillary forces during drying, significantly reducing pore collapse and making it easier to maintain an intact, interconnected porous structure. In the amorphous regions, molecular chains are entangled and disordered, hydrogen bonds are sparsely distributed, and chain segments exhibit high flexibility; the lower the crystallinity and the higher the proportion of amorphous regions, the more likely capillary forces are to cause fiber slippage, network contraction, and collapse during drying, ultimately leading to a significant decrease in material porosity and pore shrinkage.

The large number of hydroxyl groups in cellulose molecular chains can form dense intermolecular and intramolecular hydrogen bonds, thereby constructing a stable three-dimensional network structure (Figure 1b). The total number of hydroxyl groups largely determines the cross-linking strength and drying shrinkage behavior of the cellulose network: an appropriate amount of hydroxyl groups can construct a continuous three-dimensional hydrogen bond network, enhancing the integrity of the nanofiber network and reducing fiber delamination and agglomeration during drying [32]; however, an excessively high hydroxyl content makes the material overly hydrophilic, significantly increasing capillary forces during the drying stage and exacerbating pore channel compression and collapse. Therefore, regulating the extent of hydrogen bonding between cellulose molecules is the key to balancing network stability and resistance to drying shrinkage.

At the morphological level, cellulose can further exist in the form of microfibrils, nanofibers, or nanocrystals. The aspect ratio, flexibility, and entanglement capacity of fiber units at different scales significantly influence the collapse resistance of CLPMs. In general, nanofibers with high aspect ratios are more conducive to forming a continuous, interconnected three-dimensional framework, thereby enhancing the structural dimensional stability of pore networks during the drying process [31]. At the same time, the reactive hydroxyl groups at the C2, C3, and C6 positions in cellulose molecules provide abundant sites for chemical modification (Figure 1c). Introducing different functional groups through oxidation, sulfonation, or carboxymethylation can modulate inter-fiber interactions, increase fiber strength and cross-linking density, and thereby enhance structural stability after drying [33,34,35,36]. Furthermore, chemical modification can improve fiber mechanical robustness and cross-link density. For example, during the TEMPO oxidation process, the hydroxyl groups in cellulose molecules are converted into carboxyl groups, endowing the material with new chemical properties and structural characteristics, thereby enhancing hydrophilicity and laying the foundation for ionic cross-linking. In the Malaprade reaction, the ortho-diols at the C2 and C3 positions of glucose units are selectively oxidized to form 2,3-dialdehyde cellulose. This material possesses highly reactive aldehyde groups and controllable degradation properties, while also providing abundant active sites for Schiff base reactions and chemical grafting; sulfonation introduces sulfonic acid groups, endowing cellulose with excellent ion-exchange properties and heavy metal adsorption capacity.

## 3. Mechanism of Collapse During the Drying of CLPMs

The collapse of CLPMs during the drying process is not caused by a single factor, but rather results from the coupled effects of capillary pressure, hydrogen bond rearrangement, and network structural strength under different drying conditions. Essentially, as the solvent is removed, contraction stresses induced by capillary forces continuously accumulate, ultimately leading to an imbalance between capillary forces and the mechanical stability of the network structure. The ability of the cellulose framework to maintain its original pore structure depends on whether the network possesses sufficient mechanical support, an appropriate rate of hydrogen bond restructuring, and sufficiently stable interchain interactions. This section will elucidate the underlying mechanisms behind this collapse phenomenon from three perspectives, discuss the interactions among these factors, and introduce some commonly used pore characterization techniques.

### 3.1. Capillary Pressure

**Figure 2:**
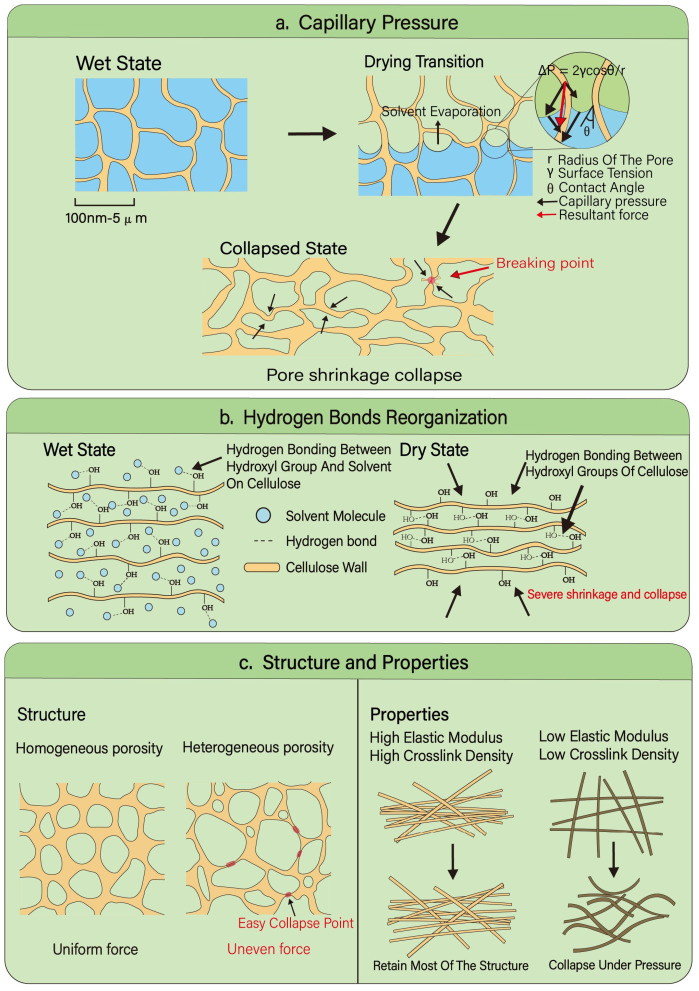
(**a**) Cellulose structural collapse induced by capillary pressure. (**b**) Cellulose structural collapse induced by hydrogen bond reorganization. (**c**) Influence of framework structural strength.

Capillary pressure is the primary external driving force that induces network contraction and collapse during ambient pressure drying. As the solvent evaporates, a gas–liquid interface forms within the gel; changes in its curvature generate capillary pressure (Figure 2a), which can be expressed by the Young–Laplace equation: ΔP = 2γcosθ/r, where γ represents the surface tension of the solvent, θ is the contact angle, and r denotes the capillary radius (pore radius) [37]. From a mechanistic perspective, for the same cellulose network, the smaller the pore size, the greater the capillary pressure, and the higher the risk of collapse [25]. As the drying process progresses, the amount of solvent within the pores gradually decreases, leading to an increase in the curvature of the gas–liquid interface and a significant rise in capillary pressure [38]. The effects of different solvents on capillary pressure vary significantly. Water has a high surface tension, so direct water evaporation is more likely to generate large contraction stresses; in contrast, low-surface-tension solvents such as ethanol and acetone can significantly reduce capillary pressure, thereby slowing the contraction of the pore walls [37,39]. Furthermore, the actual destructive effect of capillary pressure also depends on the load-bearing capacity of the network itself. When capillary pressure is lower than the network’s elastic recovery capacity, the pore structure can partially recover; however, when capillary pressure continuously exceeds the compressive strength of the framework, the pore walls undergo irreversible deformation, ultimately leading to a significant decrease in porosity and specific surface area [40,41]. Therefore, capillary pressure does not act independently; together with structural strength, it determines whether collapse occurs.

### 3.2. Hydrogen Bond Reorganization

Cellulose molecular chains are rich in hydroxyl groups, which can form both intramolecular and intermolecular hydrogen bonds; these hydrogen bonds are the foundation for maintaining the initial stability of the network. However, during the drying process, as the solvent is gradually removed, the hydrogen bonds between cellulose chains and solvent molecules weaken or even disappear, while the interchain hydrogen bonds redistribute and gradually strengthen, thereby inducing network densification [42]. This process exhibits a distinct dual effect: on the one hand, moderate hydrogen bond reorganization helps enhance pore wall strength, making the dried structure more stable; on the other hand, if hydrogen bond reorganization occurs too rapidly or to an excessive degree, it promotes the tight aggregation of cellulose chains, leading to excessive shrinkage in localized areas and subsequently causing the collapse of the pore structure (Figure 2b) [43]. In other words, hydrogen bond reorganization is both the foundation of structural stability and a potential factor inducing excessive structural shrinkage. The impact of hydrogen bond reorganization on collapse varies under different drying conditions. If the solvent evaporation rate is too fast, the molecular chains may not have sufficient time to rearrange in an ordered manner, leading to an uneven distribution of hydrogen bonds and the occurrence of localized stress concentrations. This phenomenon can trigger uneven shrinkage or even collapse of the network structure. Conversely, if the evaporation rate is too slow, it prolongs the interaction time between molecular chains but fails to promote effective hydrogen bond cross-linking, resulting in insufficient hydrogen bond formation and ultimately a loose structure. This, too, leads to insufficient structural stability and causes collapse [44,45]. Therefore, collapse is not a matter of whether faster drying is always worse or slower drying is always better, but rather depends on whether the rate of hydrogen bond reorganization matches the rate of increase in capillary forces.

### 3.3. Structural Strength

If capillary pressure is the external driving force behind collapse and hydrogen bond reorganization is the evolutionary mechanism of the internal structure, then the strength of the network structure is the key factor determining whether the material can withstand this process. The mechanical stability of cellulose gels is primarily influenced by factors such as crystallinity, crosslinking density, solid content, pore wall integrity, and fiber morphology (Figure 2c) [37,46,47,48,49]. Generally, higher crystallinity and greater crosslinking density help improve the rigidity of the framework and its resistance to deformation, thereby enhancing the material’s ability to maintain its structure during the drying process. Conversely, if the network is insufficiently crosslinked, the solid content is too low, or the pore walls are too thin, the material is more prone to plastic deformation and irreversible collapse under capillary pressure [49,50]. This indicates that the risk of collapse is determined not only by the drying process but also by whether the initial network is sufficiently strong. The geometric characteristics of the pore structure are equally critical. Uniform and continuous pore walls help disperse drying stresses and reduce local stress concentrations; in contrast, structures with uneven distribution, insufficient support, or numerous pore wall defects are more prone to collapse, which typically begins in weak areas and subsequently spreads throughout the entire network [37,48]. Furthermore, the morphology of cellulose significantly influences collapse resistance. Nanofibers with high aspect ratios are more likely to form interconnected three-dimensional frameworks, providing more continuous force transmission pathways during drying; consequently, they are generally less prone to collapse than short fibers or networks with low entanglement [51,52]. The effect of solid content cannot be overlooked. A higher solid content generally implies a higher proportion of solid framework in the network and thicker pore walls, resulting in greater resistance to collapse; however, if the solid content is too high, it may lead to an uneven pore structure in the system, thereby increasing the risk of localized shrinkage. It is evident that optimal drying results often stem from the synergistic optimization of three factors: reduced capillary pressure, controlled hydrogen bond reorganization, and enhanced network strength rather than relying on any single factor alone. Table 1 summarizes the aforementioned influencing factors.

### 3.4. Pore Characterization Techniques

To systematically evaluate the retention of pore structures in CLPMs during drying, cross-linking, modification, and service, it is necessary to combine multiple characterization techniques to analyze various aspects, including morphology, size distribution, connectivity, pore volume, specific surface area, and dynamic evolution processes. Since different techniques can observe varying pore size ranges and provide different dimensions of information, a single method often fails to fully reflect the true state of a material’s pore network; therefore, it is typically necessary to combine different techniques to achieve a comprehensive evaluation of pore collapse behavior.

#### 3.4.1. Scanning Electron Microscope (SEM)

SEM is one of the most commonly used methods for morphological characterization of porous materials. It allows for direct observation of the microstructure of material surfaces and cross-sections, and is particularly suitable for analyzing pore wall morphology, pore connectivity, framework integrity, and structural changes before and after drying. For CLPMs, SEM can clearly reveal whether honeycomb-like, fibrous, or hierarchical pore structures have undergone shrinkage, collapse, or pore wall adhesion, serving as a key basis for determining whether the macroscopic pore structure has been compromised. In practical applications, pore size uniformity, pore wall thickness, and network integrity are typically evaluated by comparing SEM images of samples before and after freeze-drying, atmospheric drying, or modification. If the sample surface exhibits obvious pore closure, framework collapse, or localized densification, this indicates insufficient pore structure stability [53]. It should be noted that SEM primarily provides two-dimensional surface information; although cross-sectional observations can indirectly reflect internal structures, its ability to reconstruct the true three-dimensional pore network is limited, and shrinkage or artifacts may be introduced during sample preparation. Therefore, SEM is more suitable as a tool for preliminary assessment of macroscopic morphology and local structural integrity.

#### 3.4.2. Transmission Electron Microscopy (TEM)

Compared to SEM, TEM offers higher spatial resolution and can be used to observe nanoscale pore wall structures, fiber cross-linking interfaces, and the dispersion of fillers within the matrix. For CLPMs containing nanopores or composite reinforcing components, TEM is particularly well-suited for analyzing whether the microstructure is uniform, whether the interfaces are tightly bonded, and whether nanoscale defects may induce localized collapse [54]. The advantage of TEM lies in its ability to reveal finer structural features within the material, such as the arrangement of cellulose microfibrils, the embedding state of nanofillers, and the continuity of cross-linking interfaces. However, TEM imposes strict requirements on sample thickness, involves a complex sample preparation process, and has a limited field of view; therefore, it is difficult to directly characterize the overall distribution of large-scale pore networks. Consequently, TEM is better suited for use in combination with other techniques to jointly verify the stability of pore structures across the nanometer-to-micrometer scale.

#### 3.4.3. Mercury Intrusion Porosimetry (MIP)

Mercury intrusion porosimetry is a classic technique for analyzing pore structure, primarily used to determine the pore size distribution, pore volume, and porosity of micrometer- to submicrometer-scale pores in materials. Its basic principle involves using mercury to penetrate pore channels under applied pressure, and then calculating pore structure parameters based on the relationship between pressure and pore diameter. For CLPMs, MIP can quantitatively reflect the degree of pore retention in materials after drying at atmospheric pressure or modification, and can be used to compare the effects of different preparation processes on pore size distribution. A key advantage of MIP is its high degree of quantification; it provides clear pore size distribution curves and cumulative pore volume data, thereby enabling the determination of whether the material has undergone pore size reduction, pore blockage, or collapse of large pores. If a decrease in total pore volume and a shift in the pore size distribution toward smaller pores are observed in treated samples, this typically indicates that the pore network has undergone contraction or rearrangement during the drying process [55]. However, MIP also has certain limitations. First, the high-pressure mercury intrusion process may cause secondary compression of soft porous materials; particularly for low-density, highly compressible CLPMs, test results may be subject to artificial influences. Second, MIP is less sensitive to closed pores and nanopores, and it assumes that pore channels are ideal cylinders, which often deviates from actual pore structures. Therefore, MIP is more suitable for the quantitative analysis of medium- and large-pore structures and should not be used alone as the sole basis for judging pore structure stability.

#### 3.4.4. Nitrogen Adsorption–Desorption Testing (BET)

The nitrogen adsorption–desorption method, particularly specific surface area testing based on BET theory, is a key method for analyzing the micropore and mesopore structures of porous materials. Through adsorption isotherms, desorption hysteresis loops, and pore size distribution models, key parameters such as the material’s specific surface area, pore volume, pore size distribution, and pore structure type can be determined [56]. For CLPMs, this technique is particularly suitable for evaluating whether a high specific surface area and an open pore network are retained after drying. BET testing typically reveals whether the material has experienced a decrease in specific surface area, a reduction in pore volume, or a loss of mesoporous structure due to pore collapse. If the material retains a high specific surface area and a reasonable pore size distribution after modification or optimization, this indicates that its pore structure has been well preserved. It should be noted that BET is more suitable for analyzing microporous and mesoporous materials, with limited capability for characterizing macroporous materials; furthermore, test results are significantly influenced by sample pretreatment, degassing conditions, and model selection. Therefore, when studying CLPMs, BET is often used in conjunction with SEM and MIP to achieve complementary analysis of pore structures from the nanoscale to the micrometer scale.

## 4. Manufacturing Strategies for Ambient Pressure Drying

As discussed above, the core challenge of CLPMs at ambient pressure lies in network collapse, structural shrinkage, and a decrease in porosity caused by capillary pressure at the solid–liquid interface during the drying process. To address this issue, researchers have developed five mature manufacturing strategies based on three key approaches: reducing capillary pressure, enhancing the mechanical stability of the network, and regulating pore structure formation. These strategies include solvent substitution, surface hydrophobic modification, the introduction of high-strength components, chemical/physical cross-linking and the ice crystal templating method. Each strategy addresses the issue of structural retention during ambient pressure drying from a different perspective, combining its own technical characteristics, performance advantages, and applicable scenarios. These strategies can be implemented individually or in combination to further enhance drying stability and the material’s overall performance, providing diverse technical pathways for the low-energy, large-scale production of CLPMs.

### 4.1. Solvent Substitution

**Figure 3:**
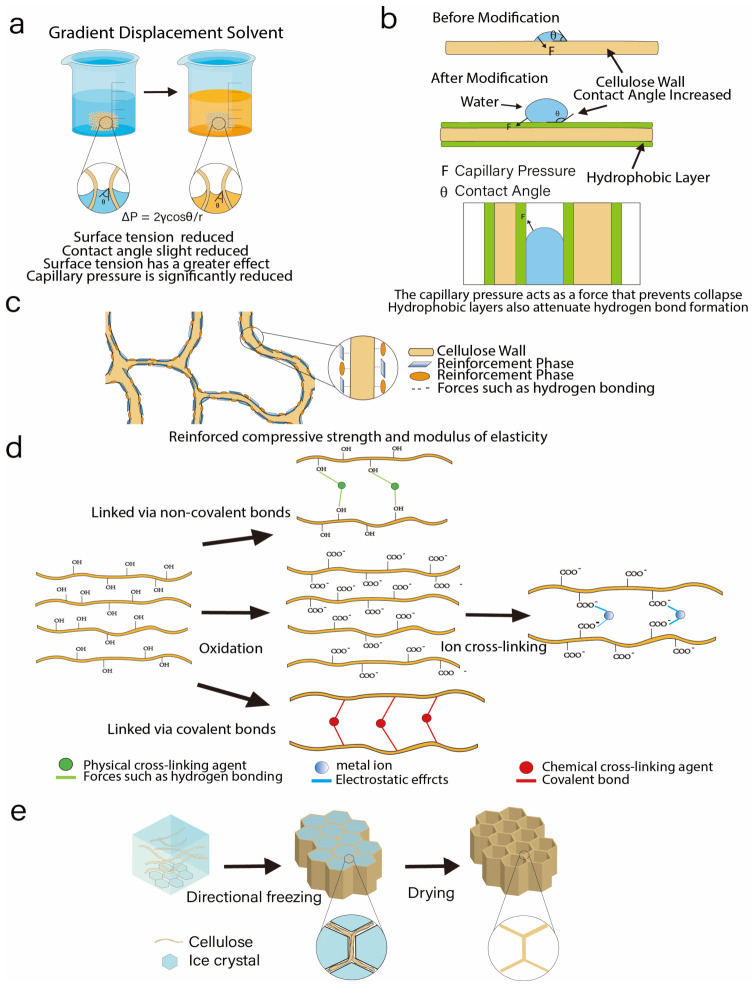
(**a**) Schematic diagram of solvent substitution. (**b**) Schematic diagram of surface hydrophobic modification. (**c**) Schematic diagram of Introduction of high-strength components. (**d**) Schematic diagram of chemical/physical cross-linking. (**e**) Schematic diagram of ice crystal template.

The solvent substitution strategy fundamentally reduces the capillary pressure at the solid–liquid interface during the drying process by replacing traditional highly polar solvents with low-surface-tension, low-polarity solvents. This minimizes the damage caused by capillary pressure to the cellulose network and ensures structural stability under ambient pressure drying conditions. Replacing water with a low-surface-tension, low-polarity solvent significantly reduces the surface tension at the solid–liquid interface. Although this substitution may slightly reduce the contact angle, the change in surface tension is more pronounced, leading to a substantial reduction in capillary pressure. Furthermore, low-polarity solvents interact with cellulose hydroxyl groups via weaker hydrogen bonds and are less likely to form strong hydrogen bond associations. This characteristic helps mitigate molecular chain aggregation and shrinkage caused by hydrogen bond reorganization during drying, thereby effectively maintaining the structural stability of the material under ambient pressure drying conditions (Figure 3a). Due to its direct mechanism of action and significant effects, this method typically yields high volume retention and pore structure integrity. However, to prevent damage to the cellulose structure caused by sudden solvent replacement, gradient replacement is commonly employed, involving sequential immersion in low-polarity solvents of gradually increasing concentration to progressively replace the water within the network, ultimately achieving complete water replacement. Commonly used low-polarity solvents include ethanol (22.4 mN/m at 25 °C) [57], tert-butanol (20.3 mN/m at 25 °C) [58], isopropanol (20.9 mN/m at 25 °C) [59,60], acetone (23.7 mN/m at 25 °C) [60], and n-hexane (17.9 mN/m at 25 °C) [61]. For example, the cellulose foam prepared by Wang’s team used ethanol to replace the original solvent, water, and was dried in an oven at 60 °C under ambient pressure. This foam achieved a maximum volume retention of 71.0%, a 91.9% improvement over direct ambient pressure drying, with a porosity of 92.3% [57]. The main advantage of this method lies in its ability to significantly reduce capillary pressure, thereby achieving excellent structural retention. After replacement with a low-polarity solvent, cellulose-based materials not only possess a large specific surface area and high porosity [57,58,60], but also effectively retain their original pore structure without compromising core properties such as adsorption capacity and thermal insulation. Furthermore, this method is simple to implement and is highly compatible with the other four ambient pressure drying methods. When used in combination with these other methods, it can significantly enhance drying stability.

However, the practical application of this strategy is limited by the number of solvent exchange cycles, the duration of each cycle, and solvent recovery efficiency: for thick samples or systems with high water content, complete replacement often requires a long time, and multi-stage solvent exchange increases process complexity and solvent consumption. Therefore, this method is more suitable for laboratory-scale preparation and small- to medium-scale production; to further enhance scalability, it must be combined with a highly efficient solvent recycling system. From an environmental impact perspective, while solvents such as ethanol, isopropanol, and acetone can reduce capillary pressure more effectively than water, they are more volatile and flammable. Without effective ventilation, closed-loop recovery systems, and fire-safety measures, their use in industrial-scale applications would pose additional safety and environmental risks. Overall, this method generally exhibits good compatibility with cellulose feedstocks from various sources and is suitable for plant-based cellulose, nanocellulose, and certain regenerated cellulose gel systems, making it one of the most versatile anti-collapse strategies.

### 4.2. Surface Hydrophobic Modification

The core principle of surface hydrophobic modification strategies is as follows: cellulose molecular chains are rich in hydrophilic hydroxyl groups; when in contact with water, the solid–liquid interfacial tension is high, and the curved liquid surface formed by solvent evaporation during the drying process generates strong capillary pressure, causing the fiber network to shrink and collapse. By introducing long-chain alkyl or silane hydrophobic groups onto the cellulose surface through hydrophobic modification, the material’s surface contact angle can be significantly increased (for example, Yang’s team reported that after modification with 3-aminopropyltriethoxysilane, the water contact angle increased to 153.2° [62]), thereby reducing capillary pressure (Figure 3b). Furthermore, hydrophobic surfaces can form a physical barrier that reduces hydrogen bonding between the solvent and the hydroxyl groups of cellulose, thereby inhibiting the aggregation and contraction of molecular chains caused by hydrogen bond reorganization and helping to preserve the porous structure after drying at ambient pressure. Commonly used modifiers include octylamine [63,64], 3-aminopropyltriethoxysilane [62], sodium dodecyl sulfate [65], and diethoxymethylvinylsilane [66]. This method does not require complex chemical reactions; the process is simple and achieves excellent structural stability. For example, the Cervin team used octylamine for surface modification followed by oven drying, resulting in a cellulose foam with an extremely low shrinkage rate, an optimal porosity of 98.0%, and a minimum porosity of 86.7% [63]. The Sankhla team, on the other hand, used diethoxymethylvinylsilane for modification to prepare a cellulose aerogel that combines mechanical strength with high porosity, achieving a porosity of 96.93% and a modulus of elasticity of 1345.4 kPa; hydrophobic modification further endowed the aerogel with excellent oil absorption properties [65]. The advantage of surface hydrophobic modification lies in its ability to simultaneously achieve material modification and collapse resistance, endowing the material with multifunctional properties: the modified CLPMs are not only resistant to drying under ambient pressure but also exhibit excellent water repellency and oil–water separation capabilities; some modifiers can also confer antibacterial and antioxidant functions.

However, the limitations of this strategy are also quite evident. First, the modification process often requires additional reagents and reaction time, making it more complex than simple solvent exchange. Second, the cost of the modifying agents, reaction efficiency, and the issue of removing residual reagents can affect its environmental friendliness. Third, some hydrophobic modifications can reduce the material’s hydrophilic responsiveness, making them unsuitable for applications requiring rapid liquid absorption or strong hydrophilic interfacial interactions. Overall, surface hydrophobic modification is more suitable for materials requiring water repellency, moisture resistance, and functional integration, rather than scenarios where the lowest cost and highest production capacity are the primary goals. It demonstrates good compatibility when used in conjunction with different cellulose raw materials and various ambient pressure drying strategies.

### 4.3. Introduction of High-Strength Components

The network structure of pure CLPMs is formed by cellulose molecular chains intertwined via hydrogen bonds, which typically results in relatively low mechanical strength. This weakness makes the material unable to withstand the capillary pressure generated during drying at ambient pressure, leading to chain slippage and structural shrinkage. The strategy for introducing high-strength components involves incorporating high-modulus, high-strength reinforcing phases into the cellulose matrix. These reinforcing phases are dispersed throughout the cellulose network as scaffold nodes, forming a stable reinforced network through mechanisms such as hydrogen bonding, covalent bonding, and van der Waals forces. This dual-function design significantly enhances the network’s overall mechanical strength and elastic modulus, providing sufficient load-bearing capacity to resist capillary pressure. Furthermore, the interfacial bonding between the reinforcing phase and cellulose helps suppress molecular chain slippage and aggregation, effectively stabilizing the porous structure. Consequently, this strategy ensures that high porosity and pore structure integrity are maintained even after drying at ambient pressure (Figure 3c).

Based on the type of reinforcing phase, this strategy can be classified into three major categories: inorganic-reinforced composites, organic-reinforced composites, and biomass-based reinforced composites. Among these, inorganic reinforcing phases are the most widely used due to their high mechanical strength and excellent thermal stability. Inorganic reinforcing phases primarily include silica nanoparticles [62], graphite [67], MXene [68], kaolin [69,70], and montmorillonite [71]. These materials possess high specific surface areas and exceptional mechanical properties; when compounded with cellulose, they can significantly enhance the compressive strength and elastic modulus of the final material. For example, adding kaolin to cellulose materials can increase the compressive strength to 0.126 MPa and the elastic modulus to 0.805 MPa [70]. Organic reinforcing phases consist primarily of polymers such as polyvinyl alcohol [72], chitosan [73], and sodium alginate [71]. Natural polymers exhibit good compatibility with cellulose and can form stable composite structures through hydrogen bonding or covalent bonding; synthetic polymers, on the other hand, enhance the flexibility of the cellulose network, thereby preventing material embrittlement. For example, composites of chitosan and cellulose aerogel form a hydrogen-bonded cross-linked structure between hydroxyl and amino groups, which not only improves drying stability but also significantly enhances the material’s thermal stability and thermal insulation durability [73]. In recent years, biomass-based reinforcing phases have become a research hotspot, primarily including components such as lignin [74,75]. These reinforcing phases are derived from abundant and renewable resources; when combined with cellulose, they enable the production of entirely biomass-based materials. Taking lignin-cellulose composites as an example, they not only improve the mechanical strength of the network structure but also enhance the material’s thermal stability—under ambient pressure drying conditions, their thermal stability is approximately 40 °C higher than that of pure cellulose materials [74]. The primary advantage of the strategy of introducing high-strength components lies in its ability to simultaneously improve both the drying stability and mechanical properties of the material, effectively addressing issues such as low mechanical strength and susceptibility to collapse that are common in pure CLPMs. This makes the material suitable for structural and functional applications with specific mechanical performance requirements. Furthermore, the introduction of reinforcing phases can also endow the material with additional functional properties, thereby expanding its potential range of applications.

From a comparative perspective, inorganic reinforcing phases typically have higher modulus and thermal stability, and can significantly improve structural retention; however, they may lead to increased material density, uneven dispersion, and adverse effects on material degradability. Organic reinforcing phases are more conducive to maintaining flexibility and processability, but the increase in strength is often limited. Biomass-based reinforcing phases offer distinct advantages in terms of sustainability, but their reinforcing effect is significantly influenced by the source of raw materials. Overall, this strategy offers good scalability, but the retention of final properties is highly dependent on the uniformity of reinforcement phase dispersion and interfacial bonding strength. Compatibility with different cellulose feedstocks is generally good; however, if the feedstock has high crystallinity or significant fiber agglomeration, the reinforcement phase cannot be uniformly distributed, thereby affecting the reproducibility and consistency of large-scale production.

### 4.4. Chemical/Physical Cross-Linking

Chemical and physical cross-linking strategies involve forming chemical cross-links or physical interactions between cellulose molecules to construct a new cross-linked network. By increasing the cross-linking density, these strategies enhance the structural stability of the cellulose network, suppress molecular chain slippage, agglomeration, and shrinkage during the drying process, and rely on the high-strength cross-linked network to resist capillary pressure. They are among the most widely used strategies in ambient pressure drying and offer the most consistent structural retention. Based on the method of cross-linking, this strategy is divided into two categories: chemical cross-linking and physical cross-linking (Figure 3d).

The core principle of chemical cross-linking lies in the use of cross-linking agents to react with the hydroxyl groups on cellulose molecular chains through various reactions, including etherification [76,77], amidation [78], Schiff base reaction [79], Michael addition [80], and acetalization [69,71]. This process forms stable covalent cross-links between the molecular chains, connecting linear or branched cellulose molecules into a three-dimensional network structure. This significantly enhances the network’s cross-linking density, mechanical strength, and resistance to shrinkage, endowing the cross-linked cellulose network with sufficient rigidity to withstand capillary pressure during ambient-pressure drying, thereby preventing structural collapse. Common cross-linking agents include glutaraldehyde [69,71,79], 4-(4,6-dimethoxy[1,3,5]triazin-2-yl)-4-methylmorpholinium chloride hydrate [78], and citric acid [81]. These reagents exhibit high cross-linking efficiency and rapidly form stable covalent bonds, thereby effectively controlling the shrinkage rate of the material during ambient pressure drying. For example, Hu et al. reported that by treating cellulose aerogel with aminopropylsilane to introduce amino groups onto the cellulose surface, followed by a Schiff base reaction with glutaraldehyde, a shrinkage rate of 3.3% could be achieved. The thermal stability and water resistance of the material were significantly improved, and the Schiff base reaction also conferred dye adsorption capacity [79].

The core principle of physical cross-linking is to establish physical cross-linking points between cellulose molecules through noncovalent interactions such as hydrogen bonding, electrostatic forces, molecular entanglement, and crystallization. This process forms a stable three-dimensional network structure without the need for chemical cross-linking agents, making the preparation process environmentally friendly and mild. Common physical cross-linking methods include the use of ionic cross-linking agents [67,82,83,84]. Metal ion cross-linking can significantly enhance the overall performance of cellulose foams: for example, samples cross-linked with Fe^3+^ achieved a compressive modulus of 1213 kPa [67]; Al^3+^, on the other hand, enhances structural rigidity and prevents significant brittle fracture during processing [82]. The primary advantage of chemical/physical cross-linking strategies lies in the extremely high stability of the cross-linked network, which ensures that the material maintains excellent structural integrity during drying at ambient pressure. The porous structure of cross-linked CLPMs is well preserved, demonstrating good structural reproducibility, making them highly suitable for large-scale production. Furthermore, this cross-linking process offers good compatibility and can be flexibly combined with technical approaches such as surface hydrophobic modification or the addition of high-strength components to further enhance the material’s drying stability and overall performance.

Chemical cross-linking typically achieves higher structural fixation efficiency and more stable dry retention rates, but it also presents issues such as high cross-linking agent costs, the need for precise control of reaction conditions, difficulty in completely removing residual reagents, and potential environmental impacts; physical cross-linking, on the other hand, is a gentler and more environmentally friendly process suitable for large-scale processing, though its structural stability and long-term durability may be inferior to those of covalent cross-linking. Overall, this strategy strikes a good balance between scalability and performance retention, making it particularly suitable for structural materials with high requirements for compressive strength and low shrinkage. However, different cellulose feedstocks respond quite differently to crosslinking agents; for example, nanocellulose typically forms a more uniform crosslinked network than ordinary fibers, while highly crystalline feedstocks may limit the number of crosslinking sites.

### 4.5. Ice Crystal Templating

The ice crystal templating method utilizes solvent ice crystals as dynamic pore-forming templates to construct oriented and ordered porous structures through a freezing process. This technique fully leverages the ordered network formed by templating to resist drying shrinkage, combining the high porosity advantages of freeze-drying with the low energy consumption characteristics of ambient pressure drying. It is an effective method for preparing high-porosity, oriented porous CLPMs (Figure 3e). The ice crystal templating method involves two key steps: (i) freezing the prepared hydrogel or wet foam; (ii) removing the ice crystals. During the freezing process, solvent molecules nucleate and grow into ice crystals. As the ice crystals grow, the cellulose components may be expelled and aggregate in the regions between the ice crystal interfaces, aligning in an oriented manner along the crystal boundaries to form a three-dimensional porous network that mimics the morphology of the ice crystals. The ice crystals can then be removed via low-temperature, ambient-pressure drying to preserve this structure [21,85]. For example, Li’s team achieved ambient-pressure drying of cellulose aerogels by combining the ice crystal templating method with ionic crosslinking and solvent replacement. The resulting aerogels exhibited significant anisotropy, with excellent mechanical compressibility in the radial direction, cyclic compression durability, and high axial mechanical stiffness [86]. Similarly, Shi’s team employed the ice crystal templating method, combined with the introduction of high-strength components, solvent replacement, and ionic cross-linking techniques, to successfully prepare cellulose-based aerogels with low shrinkage (8.5–11.3%), ultra-low density (14.6–16.4 mg/cm^3^), and excellent mechanical properties, with a specific strength of up to 10.14 kN·m/kg. These aerogels can support loads 5000 times their own weight and retain structural integrity even after 8 h of ultrasonic treatment [87]. The main advantage of this strategy lies in its ability to produce highly porous materials with a highly ordered, oriented pore structure, where the pore structure can be precisely controlled through freezing parameters; this oriented pore structure also significantly enhances the material’s thermal insulation and mechanical properties [88,89], making it suitable for applications such as efficient adsorption and oil–water separation. Furthermore, compared to traditional freeze-drying methods, this preparation process significantly reduces costs.

However, from an industrial perspective, this method involves relatively high process complexity and requires precise control of the freezing rate, temperature gradient, and subsequent drying conditions; at the same time, if the precursor system is too dilute or the cellulose network provides insufficient support, it may also lead to localized instability in the template structure during the drying stage. Furthermore, the ice crystal templating method relies more heavily on equipment and process control, so its scaling costs and energy consumption are typically higher than those of other strategies. Nevertheless, this strategy’s unique advantages in the preparation of high-performance, oriented porous materials remain evident, and it is particularly well-suited for applications with specific requirements for pore orientation and mechanical anisotropy.

### 4.6. Summary

Overall, the solvent substitution method is the most straightforward and most effective for preserving structure, but it typically involves a solvent replacement step and recovery costs; surface hydrophobic modification can simultaneously improve collapse resistance and multifunctionality, but it presents issues related to chemical modifier consumption and post-treatment of solvents and reagents; the introduction of high-strength components is more suitable for enhancing the scaffold’s load-bearing capacity, but its performance is highly dependent on the uniformity of component dispersion and interfacial compatibility; chemical/physical cross-linking can significantly improve network stability and dry retention rate, serving as a universal strategy that balances performance and structural stability, though it varies considerably in terms of reaction condition control, post-processing, and environmental impact; the ice crystal templating method is better suited for constructing oriented, ordered pore structures, particularly for high-value-added functional materials, but its process steps and process control are relatively complex. Based on these differences, Table 2 summarizes and compares the various strategies in terms of scalability, environmental impact, cost, process complexity, solvent recovery rate, performance retention, and raw material compatibility to help readers select an appropriate ambient pressure drying pathway based on their specific application scenarios.

## 5. Applications of CLPMs

CLPMs demonstrate exceptional application potential in a wide range of fields, including adsorption, thermal insulation, flame retardancy, packaging, sensing, electromagnetic shielding, and seawater desalination. Their outstanding performance stems from the following core advantages: renewability, biodegradability, low density, high porosity, large specific surface area, low thermal conductivity, and ease of surface functionalization. However, from a practical application perspective, the value of CLPMs depends not only on whether they can perform a specific function, but also on their performance limits, long-term stability, cycle durability, environmental adaptability, and scalability in specific scenarios. Overall, the maturity of CLPMs varies across different application scenarios: research in adsorption and thermal insulation is relatively more advanced, with numerous examples of performance optimization; flame retardancy, electromagnetic shielding, and solar desalination rely more heavily on composite modification and structural design; while they offer higher performance ceilings, they also impose stricter requirements on material composition and process control; packaging and cushioning materials place greater emphasis on low cost and biodegradability. Based on this, the following sections will explore the application prospects of CLPMs by examining specific application scenarios.

### 5.1. Adsorption

An ideal adsorbent must possess a high specific surface area, a porous structure, excellent stability, and outstanding mechanical properties, while also being naturally green, renewable, and recyclable. CLPMs feature a three-dimensional, interconnected porous network structure that combines high porosity with a large specific surface area, providing ample adsorption sites. As the primary component, cellulose is abundant and biodegradable; its molecular chains are rich in hydroxyl groups and other reactive functional groups, which facilitate surface modification. Furthermore, its overall structure is stable, it possesses good mechanical properties, and it is resistant to acidic and alkaline environments, making it highly suitable for complex adsorption applications. Therefore, CLPMs have reached a high level of maturity in the field of adsorption and are particularly well-suited for applications such as wastewater treatment and oil–water separation.

**Figure 4:**
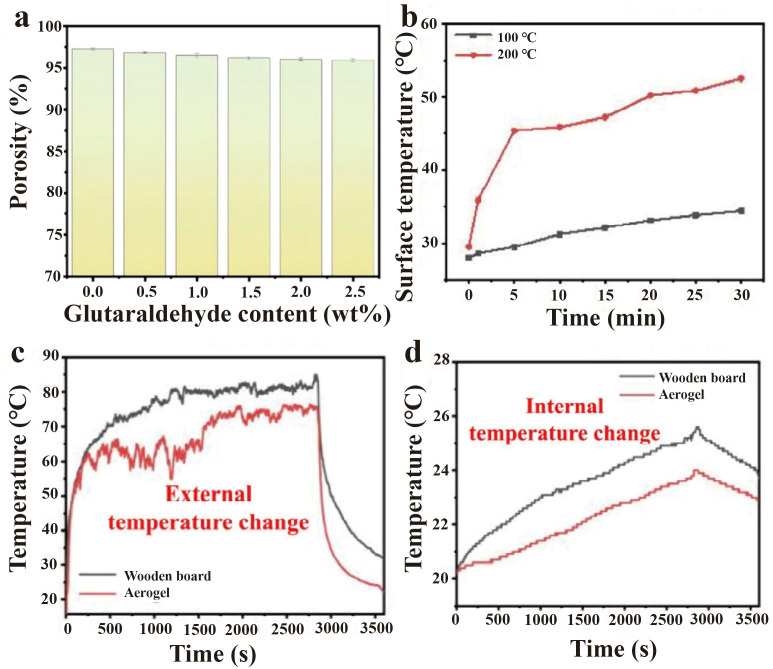
(**a**) Porosity of aerogels prepared by Zhang’s team with different glutaraldehyde contents [95]. (**b**) Temperature–time curves of aerogel heated on the hot platform [95]. (**c**,**d**) External and internal temperature–time curves after exposure and nonexposure to infrared irradiation under the wood and aerogel [95].

In the field of heavy metal ion adsorption, the hydroxyl and carboxyl functional groups in cellulose molecules can undergo chelation or ion exchange reactions with heavy metal ions, thereby effectively purifying wastewater containing heavy metals. Furthermore, chemical modifications such as oxidation or aminization can introduce additional active functional groups, further enhancing adsorption capacity and selectivity [90]. For example, the cellulose aerogel prepared by Li’s team (Figure 4a) exhibited excellent sensitivity and selectivity toward Cr^6+^ under ambient-pressure drying conditions, with a detection limit as low as 1.12 μM and a linear range of 5–50 μM; at room temperature, the adsorption capacity for Cr^6+^ can reach 166.25 mg/g, and its adsorption performance can be directly observed with the naked eye (Figure 4b,c), far exceeding that of most cellulose aerogels prepared by freeze-drying. It also demonstrates exceptional stability; after five adsorption–desorption cycles, the material still retains excellent adsorption performance, exhibiting good stability and adsorption characteristics [91]. Furthermore, CLPMs, with their cellulose framework and porous structure, exhibit significant removal efficiency for microplastics in water: the cellulose foam developed by Feng’s team achieved a filtration efficiency of up to 99.4% and a flux of 7257 L/(m^2^·h); simultaneously, the abundant hydrogen bonding sites and active functional groups in the foam matrix endow it with an excellent microplastic adsorption capacity of up to 720.4 mg/g. After 12 cycles, the stress–strain curves of the foam remained virtually identical, indicating that the foam’s structure remains stable during long-term cycling. Its production cost is significantly lower than that of comparable materials, demonstrating broad application prospects [92]. Regarding the adsorption of organic pollutants, surface modification of CLPMs can significantly enhance their adsorption efficiency for oils, organic solvents, and other organic pollutants. The cellulose aerogel developed by Sankhla’s team exhibited an oil adsorption capacity of 24 g/g, while its adsorption capacity for organic solvents such as dichloromethane and chloroform exceeded 28 g/g [66]. The cellulose foam developed by Montazeri’s team can rapidly adsorb a variety of oils and organic solvents, with adsorption capacities ranging from 10.8 to 13.6 g/g, and can be reused up to 10 times. Furthermore, compared to natural cellulose fibers, its thermal stability is significantly improved, with a water contact angle of 137.47°, providing an effective solution for efficient oil–water separation [93].

However, several challenges remain before this field can achieve large-scale commercialization, such as adsorption sites are easily masked under conditions of high salinity, the presence of complex organic compounds, or repeated use; actual capacity is often lower than that observed under ideal laboratory conditions; and the degree to which the modification process can be made environmentally friendly is limited. Future research should focus on applicability in real wastewater, adsorption kinetics, and cycle stability.

### 5.2. Thermal Insulation

Traditional building insulation materials (such as polystyrene, polyurethane, and polyethylene) generally have significant drawbacks: they are not biodegradable, place a heavy burden on the environment, and may contribute to plastic pollution; therefore, they are not suitable for green and sustainable development [3]. In contrast, CLPMs, composed of natural, renewable cellulose, are fully biodegradable and environmentally friendly. They also feature low thermal conductivity, high porosity, and excellent thermal and chemical stability, making them highly efficient thermal insulation materials. They represent one of the most mature application areas currently closest to industrial-scale production. Their structure forms a continuous three-dimensional porous network capable of effectively trapping large volumes of stationary air; since air has an extremely low thermal conductivity, this structure naturally suppresses convective heat transfer, significantly reducing overall heat conduction efficiency. Furthermore, the cellulose matrix possesses exceptional heat resistance and structural stability, maintaining resistance to shrinkage or collapse even under high-temperature or long-term use conditions, thereby ensuring durable and stable thermal insulation performance. For example, the cellulose foam developed by Sun’s team has a porosity of 97.3% and a thermal conductivity of 40.3 ± 0.6 mW·m^−1^·K^−1^, with thermal insulation performance comparable to that of commercially available insulation materials such as mineral wool and polystyrene. This foam material has a compressive modulus of 1211.5 ± 60.6 kPa, excellent moisture stability, and biodegradability (with a weight loss of 91.6% within 30 days in soil), while its porous structure remains intact in humid environments and its thermal conductivity does not increase significantly upon water absorption, making it a high-performance, environmentally friendly alternative material suitable for applications requiring mechanical strength, thermal insulation, and safety, such as vibration damping and building insulation [94].

The cellulose aerogel prepared by Guo’s team has a porosity as high as 95.8% and a thermal conductivity as low as 34.5 mW·m^−1^·K^−1^ (Figure 4a). After 30 min of continuous heating on a constant-temperature test bench at 100 °C and 200 °C, the temperature difference between the aerogel and the heating bench reached 65.6 °C and 147.5 °C, respectively. Under the same heat source, the temperature rise inside the chamber was significantly lower than that of wooden insulation panels, effectively blocking heat transfer over the long term; the porous framework suppresses heat conduction, and the carbon residue is higher during high-temperature thermal decomposition, ensuring structural stability under high-temperature conditions. Compared to conventional biomass insulation materials, it offers outstanding thermal insulation advantages and is suitable for long-term thermal insulation applications in buildings and equipment (Figure 4b–d) [95]. The core challenges currently facing this field primarily include: limited resistance to wet-heat aging during long-term service, decreased structural stability under high-humidity or temperature-cycling conditions, the current necessity to incorporate inorganic fillers, and the need to further balance cost and performance. Future efforts should focus on systematic optimization centered on low thermal conductivity, high strength, resistance to wet heat, and scalable production.

### 5.3. Flame Retardant

**Figure 5:**
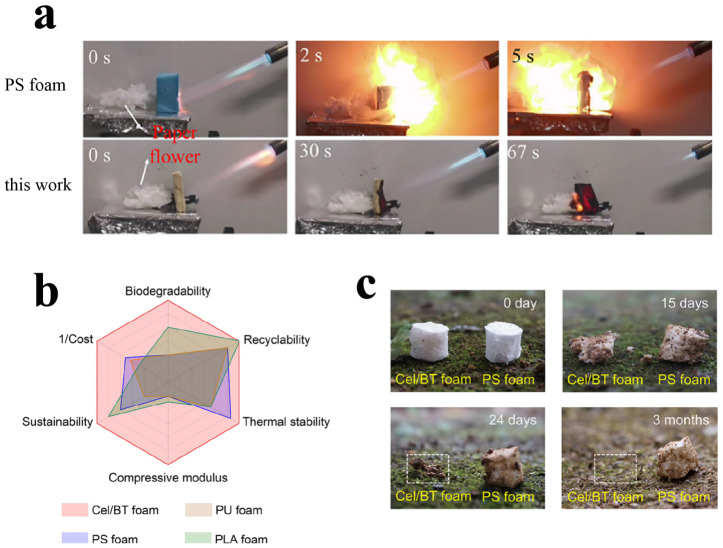
(**a**) Comparison of flame retardancy between cellulose foam prepared by Zhang’s team and PS foam [96]. (**b**) Comparison of this cellulose foam prepared by Chen’s team (Cel/BT foam) with three other common foam materials in terms of biodegradability, recyclability, thermal stability, compressive modulus, sustainability, and cost, including PS foam, PU foam, and polylactic acid (PLA) foam [97]. (**c**) Biodegradability tests of the Cel/BT foam and the commercial plastic PS foam under soil environment [97].

Effective flame-retardant materials should possess properties such as fire resistance, low thermal conductivity, self-extinguishing characteristics, and excellent thermal stability. CLPMs inherently exhibit good thermal stability, and their unique porous network structure significantly reduces thermal conductivity, effectively inhibiting heat transfer. When cellulose is heated, rapid dehydration and carbonization reactions occur, forming a dense protective carbon layer on the surface that isolates heat and oxygen, thereby conferring exceptional self-extinguishing properties to the material. Furthermore, the abundant closed micropores in the material’s structure impede internal heat convection, further enhancing its flame-retardant performance. The cellulose surface is rich in active hydroxyl groups, making it easy to chemically modify with flame-retardant functional groups such as phosphate esters, amino esters, and borate esters, thereby enhancing the flame-retardant properties of CLPMs. Consequently, CLPMs have gradually progressed from the basic functional validation stage to the performance optimization and small-scale production stage in the field of flame retardancy; however, their overall maturity remains lower than that of thermal insulation and adsorption applications. For example, the cellulose foam developed by Zhang’s team, which incorporates boronate groups, exhibits excellent flame-retardant properties: at 25 °C, its thermal conductivity is 63.4 mW·m^−1^·K^−1^, and its limiting oxygen index is 30.2%, meeting the UL-94 V-1 flame-retardant rating (Figure 5a). This material remains stable in high-temperature flame environments up to 1300 °C, effectively preventing flame spread [96]. Additionally, the material reported by Yang’s team—modified with KH550 and silica nanoparticles—exhibited a significantly increased initial thermal decomposition temperature. In cone calorimeter tests (heat flux of 35 kW/m^2^), both the ignition time and the time to peak heat release rate of the samples were prolonged, while the peak heat release rate decreased to 51.41 kW·m^−2^, indicating a 33.5% reduction in total heat release compared to unmodified samples. Both the combustion intensity and total heat release were significantly reduced. In alcohol lamp combustion tests, the unmodified conventional gel was flammable and burned continuously, whereas the modified gel showed no noticeable combustion, fully demonstrating its excellent flame-retardant properties [62].

However, because the flame-retardant capabilities of unmodified CLPMs are still insufficient for commercial applications, flame-retardant modification is essential. Challenges include the lack of environmental friendliness in some flame-retardant modifiers, trade-offs between flame retardancy and mechanical/thermal insulation properties, and the need to verify long-term stability under high-temperature conditions. Future efforts should prioritize the development of flame-retardant systems that are low-toxicity, recyclable, and more compatible with cellulose networks, while strengthening the synergistic design of multiple properties.

### 5.4. Packaging

Traditional packaging cushioning materials (such as expanded polystyrene, expanded polyethylene, and polyurethane foam) are primarily made from petroleum-based raw materials; they are non-biodegradable and cause significant environmental pollution [98]. In contrast, CLPMs fully meet the core requirements for packaging—providing cushioning performance, lightweight properties, and environmental benefits. CLPMs have extremely high porosity, with the majority of their volume consisting of air; consequently, their bulk density is far lower than that of solid materials. Furthermore, when subjected to external impact, the porous internal structure of CLPMs undergoes staged compression and deformation, effectively absorbing and dispersing impact energy, thereby achieving cushioning and shock-absorbing effects. Because CLPMs are inherently well-suited for use as packaging cushioning materials, they have reached a high level of application maturity. More importantly, the excellent biodegradability of CLPMs offers broad application prospects in food packaging, electronics packaging, and logistics. For example, the Itkor team developed a cellulose foam suitable for food packaging using citric acid cross-linking technology, providing an alternative to traditional petroleum-based plastic foams; the thermal insulation performance of this foam rivals that of commercial insulation materials and fully meets the temperature maintenance requirements for food cold-chain transportation. Its mechanical properties are comparable to those of bio-foams produced via freeze-drying and commercial plastic foams, providing excellent cushioning protection and demonstrating broad prospects in thermal insulation packaging for the food cold chain and general food cushioning applications [99]. Su’s team further optimized the cushioning performance of the cellulose-based foam: the foam features low density (with a modulus of elasticity ranging from 77 to 501 kPa), a maximum compressive strength of 183 kPa, a relatively low cushioning coefficient (3.0), and high energy absorption efficiency (32.8%). Its cushioning performance surpasses that of most commercial petroleum-based foams (such as polystyrene and polyethylene foams), making it an ideal eco-friendly alternative to traditional petroleum-based cushioning packaging materials. It effectively absorbs impact energy and protects packaged products from collision damage [100].

Furthermore, composite modification techniques can further enhance the performance of cellulose materials and expand their application scenarios. For example, the cellulose composite foam developed by Han’s team achieves dual optimization of protective performance and environmental sustainability, its compressive strength is increased by 207% compared to pure cellulose foam, reaching 28.33 kPa, and after 100 cycles of 50% compression, the foam exhibited a strain loss of only 28.39%. It retained its framework structure even after repeated compression, demonstrating long-term durability under humid and cyclic loading conditions. At the same time, it possesses excellent cushioning capabilities to protect food from impact damage during transportation, as well as flame-retardant and thermal insulation properties [101]. The foam prepared by Chen’s team exhibits an 80% strain compression modulus of 39.4 MPa, with cushioning performance far superior to that of commercial plastic foams such as PS and PU (Figure 5b), it remains structurally stable after three months of exposure to wet and dry conditions as well as outdoor storage, with minimal strength decay after repeated compression, making it suitable for cushioning and protecting fragile items, it is fully recyclable in a closed-loop system and completely biodegrades in soil within three months, making it a green alternative packaging material with low environmental impact (Figure 5c) [97]. Nevertheless, the practical adoption of CLPMs for packaging still requires addressing issues such as cost control, batch consistency, moisture resistance, and long-term storage stability. Future efforts should focus on developing low-cost continuous production processes, synergistic modification using biodegradable coatings, and customized structural designs tailored to different packaging grades.

### 5.5. Electromagnetic Shielding

**Figure 6:**
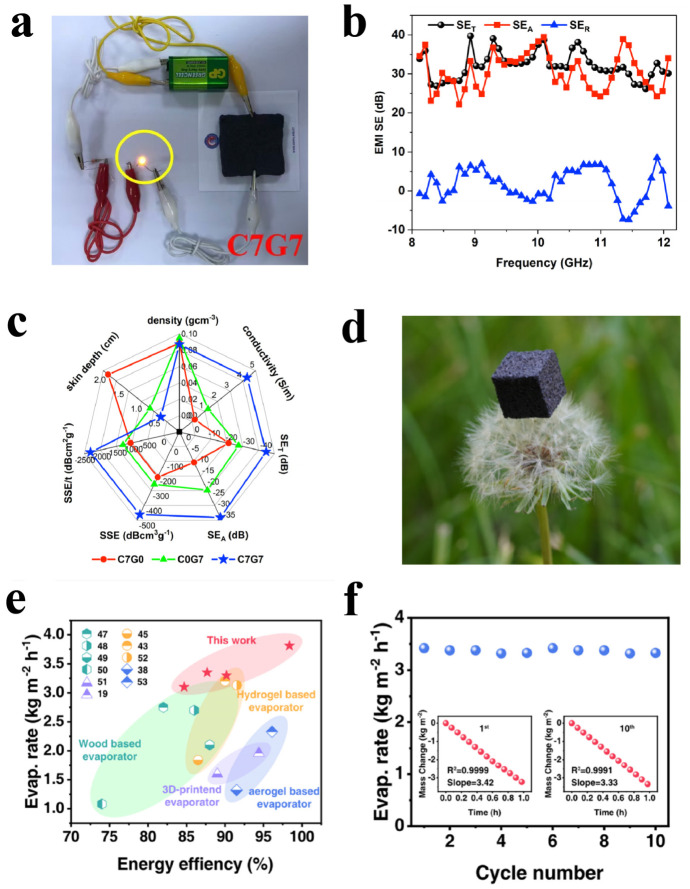
(**a**) Circuit constructed from aerogel fabricated by Taymaz’s team (The LED lamp circled in the figure is illuminated, indicating that the aerogel exhibits electrical conductivity) [102]. (**b**) Variation in electromagnetic interference total SE, absorption SE and reflection SE as a function of frequency for the aerogel fabricated by Taymaz’s team [102]. (**c**) Radar chart for comparison of density, porosity, total SE, absorption SE, skin depth, SSE and SSE/t of aerogels with different ratios prepared by Taymaz’s team [102]. (**d**) Digital photo of the aerogel fabricated by Zong’s team on a dandelion [104]. (**e**) The evaporation rate and energy efficiency of the aerogel versus other reported evaporators [104]. (**f**) Evaporation rates for 10 consecutive replicate tests of this aerogel under 1 sun irradiation [104].

With the rapid development of 5G communications, the Internet of Things and flexible electronics, the widespread adoption of various electronic devices has led to growing concern regarding electromagnetic radiation and interference. Electromagnetic pollution not only affects the operational stability of electronic devices but also poses a threat to human health. Consequently, the demand for high-performance electromagnetic shielding materials has become particularly urgent. An ideal electromagnetic shielding material should possess high shielding efficiency, excellent electrical and magnetic conductivity, low density, good mechanical stability and a configurable structure, whilst simultaneously meeting requirements for aging resistance, corrosion resistance and environmental sustainability. Thanks to their high porosity and porous network structure, CLPMs provide abundant binding sites for functional fillers, making them ideal matrix materials for electromagnetic shielding. This structure causes electromagnetic waves to undergo multiple reflections, scattering, and refraction within the channels, thereby lengthening the propagation path and enhancing the electromagnetic attenuation effect. The cellulose surface is rich in active hydroxyl groups, which can bind tightly with conductive fillers to form a conductive network, effectively increasing conductive and polarization losses. Furthermore, CLPMs are ultra-lightweight and possess excellent mechanical buffering properties and chemical stability, fully aligning with the trend towards developing lightweight, environmentally friendly electromagnetic shielding materials; however, they currently remain at the laboratory research and development stage. For example, the cellulose aerogel prepared by Taymaz’s team has been used to construct circuits (Figure 6a); this sample achieved a maximum electromagnetic interference shielding efficiency (SE) of 40.2 dB in the X-band (8.2–12.4 GHz), significantly exceeding the commercial standard of 20 dB (Figure 6b), with absorption-dominated shielding being the primary mechanism of action. At the optimal composition ratio, these aerogels exhibit an outstanding specific shielding efficiency (SSE) of 461.95 dB·cm^3^·g^−1^ and an absolute shielding efficiency (SSE/t) of 2309.29 dB·cm^2^·g^−1^ (Figure 6c) [102]. Furthermore, Wei’s team employed a dual-crosslinking composite modification strategy to successfully develop aerogels that combine high-efficiency electromagnetic interference shielding performance with environmental stability, making them suitable for a variety of electromagnetic protection scenarios: The optimized aerogel achieved an electromagnetic interference shielding performance of 62.65 dB, with an SSE value of 584.98 dB·cm^3^·g^−1^, capable of shielding over 99.99% of incident electromagnetic waves and effectively blocking electromagnetic radiation generated by devices such as Tesla coils and computers. It is worth noting that even under high-temperature conditions, this material retains excellent shielding performance of up to 59.73 dB, effectively addressing issues commonly associated with traditional biomass aerogels, such as hygroscopicity and poor environmental adaptability. This makes cellulose-based aerogel an ideal candidate material for applications in flexible electronic devices, the protection of communications equipment, and everyday electromagnetic radiation shielding [103].

Currently, this field still faces challenges such as insufficient stability of the conductive network, performance degradation in hot and humid environments, poor dispersion uniformity of fillers, and the difficulty of balancing shielding performance with flexibility and lightweight design. Future research should focus on developing more stable strategies for constructing conductive networks, low-load, high-efficiency shielding structures, and continuous manufacturing methods suitable for integration into flexible devices.

### 5.6. Seawater Desalination

Globally, 2 billion people face freshwater shortages. Solar-powered interfacial evaporation desalination technology has emerged as a leading method due to its low energy consumption, zero emissions and sustainability. High-efficiency solar interfacial evaporation materials must possess excellent hydrophilicity, high photothermal conversion efficiency, low thermal conductivity, and good salt tolerance and resistance to seawater corrosion. CLPMs fully meet these requirements: the cellulose molecular chains are rich in hydroxyl groups, endowing the material with exceptional hydrophilicity; its porous network structure enables continuous capillary transport of seawater to the evaporation interface; the low thermal conductivity of CLPMs effectively reduces heat loss to the seawater below, concentrating heat at the evaporation interface and significantly enhancing the utilization efficiency of solar thermal energy; its hierarchical porous structure promotes the reverse diffusion and reflux of concentrated salt solutions, preventing the accumulation of salt crystals on the evaporation surface that would lead to pore blockage, thereby conferring excellent salt stability on the material; whilst cellulose’s robust chemical stability enables it to withstand corrosion from high-salinity seawater, ensuring long-term performance [104,105,106]. However, the field as a whole is currently in a transitional phase from laboratory validation to application optimization. For example, the cellulose aerogel developed by Professor Zong’s team achieves an evaporation rate of 3.58 kg·m^−2^·h^−1^ under sunlight, with an energy efficiency as high as 97.2%; its photothermal conversion and water transport performance both outperform most biomass-based solar evaporators. Although the evaporation rate decreases slightly with increasing salinity, it remains stable at 2.96 kg·m^−2^·h even at a high salinity of 25%; during a continuous 10 h uninterrupted seawater evaporation cycle test, the evaporation rate remained stable [105]. Furthermore, the team prepared a cellulose/graphite composite aerogel through a modification process (Figure 6d), further enhancing its performance: under one sun irradiation, the evaporation rate reached 3.8 kg·m^−2^·h^−1^ (Figure 6e), with a photothermal conversion efficiency as high as 98.4%. This composite material exhibits excellent solar absorption capacity, photothermal conversion efficiency, rapid water transport properties and salt resistance. Following 10 consecutive seawater cycle tests, it demonstrated resistance to salt precipitation and stable evaporation performance in long-term saline environments (Figure 6f); its energy utilization efficiency far exceeds that of most biomass-based photothermal evaporation materials, significantly enhancing the economic viability and practical application value of solar seawater desalination [104]. It is worth noting that, as demonstrated by Takur’s team, the preparation process for CLPMs is cost-effective; the cellulose composite foam material they developed achieves a solar spectral absorption rate of up to 92.18% and a peak evaporation efficiency of 360%, with both freshwater yield and energy utilization efficiency significantly surpassing those of traditional tubular solar distillation systems. The raw materials are derived from renewable cellulose and air pollutants such as flue dust, and the production process is environmentally friendly, low-carbon and simple to operate. Under outdoor conditions, these foam materials can produce up to 6.4 liters of fresh water per square meter per day at a cost as low as USD 0.009 per liter, making them highly suitable for meeting drinking water needs in areas with poor infrastructure, whilst meeting the requirements for low-cost, sustainable green development [107].

However, challenges remain in this field, including striking a balance between evaporation efficiency and long-term salt tolerance; insufficient understanding of the effects of pollutants and biofouling under real seawater conditions; and concerns regarding whether thermal management and water transport uniformity will deteriorate upon device upscaling. Future efforts should focus on high throughput, resistance to salt blockage, resistance to fouling, and low-cost production.

## 6. Summary and Outlook

As a class of renewable, biodegradable, and eco-friendly materials, CLPMs exhibit broad application prospects in fields including environmental remediation, building insulation, advanced packaging, flexible electronics, and seawater desalination, owing to their low density, high porosity, large specific surface area, and ease of functionalization. They are expected to become important alternatives to traditional petroleum-based porous materials and certain inorganic porous materials. In particular, breakthroughs in ambient pressure drying preparation technologies have laid critical groundwork for the low-energy, low-cost, large-scale manufacturing of CLPMs. To date, five representative strategies have been developed for preparing CLPMs via ambient pressure drying: solvent substitution, surface hydrophobic modification, introduction of high-strength components, chemical/physical cross-linking, and ice crystal templating. By reducing capillary pressure, enhancing network stability, and regulating hydrogen bond reorganization, these strategies address the core challenge of structural collapse during ambient pressure drying and achieve synergistic optimization of both material performance and preparation efficiency.

Nevertheless, the industrial application of CLPMs still faces several key bottlenecks. First, some solvent-substitution processes rely on highly volatile and flammable organic solvents such as acetone and tert-butanol, and the solvent recovery process also incurs additional energy consumption and costs. Second, some existing hydrophobic modifiers still pose toxicity or non-recyclability issues, and chemical modification often involves the use of organic solvents, which to some extent undermines the environmental sustainability of the process. Furthermore, if high-strength components are unevenly dispersed, they can easily cause stress concentrations within the composite network, thereby reducing the material’s mechanical reliability; meanwhile, certain inorganic reinforcing phases (such as MXene and SiO_2_) may also affect the material’s biodegradability. Regarding cross-linking strategies, while chemical cross-linking is effective, most cross-linking agents still rely on organic reagents, whereas physical cross-linking systems may suffer from insufficient durability. The ice crystal template method is constrained by a relatively long preparation cycle, sensitivity to freezing parameters, and difficulties in controlling pore structure uniformity; particularly during scale-up, it is more prone to uneven pore size distribution and localized cracking.

Looking ahead, research on CLPMs should shift its focus from achieving manufacturability to ensuring scalability, sustainability, and long-term serviceability. In terms of drying technology, there is an urgent need to develop ambient pressure drying routes that minimize solvent usage, optimize solvent replacement and recovery processes, and reduce energy consumption and environmental impact. In terms of chemical cross-linking and hydrophobic modification, priority should be given to exploring bio-based cross-linking agents and recyclable hydrophobic modifiers to reduce the use of toxic reagents and irreversible chemical consumption. Regarding manufacturing processes, future efforts should focus on advancing continuous, automated, and scalable processing methods to enhance the consistency and economic viability of mass production. At the same time, hierarchical pore design based on material structural characteristics is a key direction for raising the upper limit of performance: by constructing a hierarchical structure where micropores, mesopores, and macropores work in synergy, it is possible to balance low density, mechanical strength, and mass transfer efficiency, further expanding their application potential in scenarios such as adsorption, thermal insulation, shielding, and evaporation. Research into the mechanisms of interfacial and structural stability is also a key area for future breakthroughs, aimed at developing more effective anti-collapse strategies. If breakthroughs are achieved in these fundamental issues, CLPMs can truly transition from laboratory performance validation to stable, green, and scalable industrial applications.

## Figures and Tables

**Table 1:** This table summarizes the effects of various factors on the structural collapse of cellulose.

Factors Affecting	Mechanism of Action	Effects on Capillary Pressure/Network Strength	Risk of Collapse
Surface Tension of Solvents	Determines the magnitude of capillary pressure at the drying interface	The lower the surface tension, the lower the capillary pressure	The lower the surface tension, the lower the risk
Pore Size	Affects capillary pressure	Smaller pores are more likely to generate high capillary pressure	The smaller the pore size, the higher the risk
Solid Content	Determines the initial framework density and pore wall thickness	High solid content typically enhances load-bearing capacity; excessively high levels may lead to structural inhomogeneity	The higher the solid content, the lower the risk (but excessively high levels may increase it)
Cross-linking Density	Enhances interchain connections and mechanical support	The more thorough the cross-linking, the more stable the structure	The higher the cross-linking density, the lower the risk
Hydroxyl Density	Affects hydrogen bond reorganization during drying	More hydroxyl groups result in stronger reorganization, which may stabilize the structure or cause shrinkage	The higher the density, the lower the risk (though excessively high levels may increase risk)
Nanofiber aspect ratio	Affects network formation and structural stability	A high aspect ratio facilitates the formation of a continuous framework	The higher the aspect ratio, the lower the risk

**Table 2:** Comparative summary of five ambient-pressure drying strategies for CLPMs.

Strategy	Mechanism of Action	Representative Additives/Treatments	Key Advantages	Key Disadvantages	Scalability	Solvent Recovery Requirements	Typical Applications
Solvent substitution	Replacing water with a low-surface-tension solvent significantly reduces capillary pressure	Ethanol, tert-butanol, isopropanol, acetone, n-hexane	Good pore structure retention, high volume retention rate, and a mature process	Requires multiple replacement cycles, is time-consuming, and involves high solvent consumption and recovery costs	High	Requires efficient closed-loop recovery	Thermal insulation, adsorption, lightweight materials
Surface hydrophobic modification	Increases the contact angle, reduces liquid-solid interactions, and inhibits hydrogen bond reformation	octylamine, 3-aminopropyltriethoxysilane, sodium dodecyl sulfate, and diethoxymethylvinylsilane	Simultaneously imparts hydrophobicity and collapse resistance, enabling multifunctionality	Requires additional reagents and post-treatment; some modifiers are relatively expensive	Medium	Medium	Oil–water separation, moisture-proof packaging, hydrophobic materials
Introduction of high-strength components	Constructing a reinforced skeleton using rigid fillers to improve load-bearing capacity	MXene, kaolin, polyvinyl alcohol, chitosan, sodium alginate, lignin	The higher the solid content, the lower the risk (but excessively high levels may increase it)	Significant improvement in mechanical properties and enhanced structural retention Uneven dispersion can weaken the effect and may increase density	High	Not applicable or low	Structural materials, thermal insulation and load-bearing, functional composites
Chemical/physical cross-linking	Fixing a three-dimensional network through covalent/non-covalent cross-linking to suppress segment slippage	glutaraldehyde, citric acid, ionic cross-linking agents	High structural stability, low drying shrinkage, and good reproducibility	Chemical cross-linking may leave residues and pose an environmental burden, requiring strict process control	High	Chemical cross-linking is higher, physical cross-linking is lower	High-strength aerogels, adsorption, thermal insulation, and structure-function integrated materials
Ice crystal templating	Oriented pores are formed through ice crystal growth, and the ice crystals are then removed to preserve the structure	Freezing process	The higher the density, the lower the risk (though excessively high levels may increase risk)	The process is complex, sensitive to freezing rates and temperature gradients, and requires sophisticated equipment	Low	Low	Oriented thermal insulation, adsorption, oil–water separation, high-end functional materials

## Data Availability

No new data were created or analyzed in this study. Data sharing is not applicable to this article.

## Abbreviations

The following abbreviations are used in this manuscript:

CLPMs	cellulose-based lightweight porous materials
PS	polystyrene
SEM	scanning electron microscope
TEM	transmission electron microscopy
MIP	mercury intrusion porosimetry
BET	nitrogen adsorption–desorption test
PU	polyurethane
PLA	polylactic acid
SE	shielding effectiveness
SSE	specific shielding effectiveness
